# *Curtobacterium aetherium* sp. nov., a polyextremophilic plant pathogen isolated from the stratosphere

**DOI:** 10.1128/spectrum.01774-24

**Published:** 2025-04-02

**Authors:** Jovana Mijatović Scouten, Amy Smith, Adam J. Ellington, Noelle C. Bryan, Robert M. Harveson, Brian H. Kvitko, Brent C. Christner

**Affiliations:** 1 Department of Botany and Plant Pathology, Oregon State University 170397 , Corvallis, Oregon, USA; 2 Department of Plant Pathology, University of Georgia 1355 , Athens, Georgia, USA; 3 Department of Microbiology and Cell Science, University of Florida 3463 , Gainesville, Florida, USA; 4 Department of Cardiac Surgery, Brigham and Women’s Hospital, Boston, Massachusetts, USA; 5 Department of Plant Pathology, Panhandle Research and Extension Center, University of Nebraska, Scottsbluff, Nebraska, USA; University of Mississippi, University, Mississippi, USA

**Keywords:** extremophile, aerobiology, *Curtobacterium*, plant pathogens

## Abstract

Although the high-altitude limit for microbial survival in the Earth–atmosphere system has remained a scientific curiosity and topic of study, the ecological significance of long-distance microbial dispersal in the atmosphere has been perceived to have marginal relevance. Here, we report the characterization of novel plant pathogenic species of *Curtobacterium* that were isolated from samples collected at altitudes ranging from 1.5 to 29 km above sea level. Whole genome-based phylogenies of three strains, paired with plant challenge assays, indicate that each is a previously unrecognized species and causes disease on beans comparable to *Curtobacterium flaccumfaciens*. Isolates from the stratosphere (strain L6-1) and agricultural millet (G77) were identified to be the same species and designated as *Curtobacterium aetherium* sp. nov. *C. aetherium* displays high levels of tolerance to desiccation and UV radiation, which are stresses that increase in intensity with altitude. Back trajectory air mass analysis implied that the phytopathogens may have had an intercontinental source, but regional origins in the continental US cannot be excluded. The environmentally robust phytopathogens we have documented in the upper atmosphere provide new perspective on the role that high-altitude transport may play in microbial dispersal, gene flow, and the epidemiology of aerially dispersed plant disease.

**IMPORTANCE:**

Enormous quantities and varieties of microorganisms are continually aerosolized and transported in the atmosphere, yet there is a limited understanding of the consequences to downwind ecosystems. While studying bacteria that survive extreme conditions at altitudes up to 29 km in the atmosphere, we discovered new species that have the capacity to cause disease in agriculturally relevant bean varieties. The hardiest isolate we characterized from the stratosphere is a member of the same species as an isolate from an agricultural source, which we have designated *Curtobacterium aetherium*. *C. aetherium* is a phytopathogen capable of enduring high-altitude and long-distance atmospheric transport while also possessing the potential to opportunistically infect crops in deposition locations.

## OBSERVATION

The atmosphere is an important medium for biological dispersal, including the rapid dissemination of communicable diseases. Due to their aerodynamic diameters, bacteria typically have residence times in the lowest layer of the atmosphere (i.e., the troposphere) that range from days to weeks (1), durations that are sufficient to be transported thousands of kilometers from their sources (2). Given that bacteria are aerosolized from virtually all terrestrial and marine surface environments on Earth, the rich variety of species detectable in a given air mass is linked to its history and may represent a combination of taxa originating from local, regional, and even intercontinental sources (3). Long-range dispersal of plant pathogens has mostly been explored in the context of obligately biotrophic rust fungi, where airborne dispersal of their environmentally durable spores has been documented on continental to global scales (4). While the aerodynamic properties of bacteria are well suited for long-range and recurrent transport in the atmosphere, the processes of aerosolization and atmospheric transport are physiologically stressful and unfavorable to survival (3). Hence, bacterial phytopathogens tolerant to a combination of environmental stresses, including water deficit and ultraviolet radiation (UVR), would have the highest potential to survive long-distance airborne dissemination to new hosts. Importantly, these stresses intensify with altitude into the stratosphere, where environmental conditions and long air residence times are thought to limit survival of even the hardiest bacteria (5).

Previously, we reported bacterial isolates that were cultured from aerosol samples collected at altitudes up to 29 km above sea level (6, 7) using a helium balloon platform and aerosol-sampling payload system (8). HYbrid Single Particle Lagrangian Integrated Trajectory (HYSPLIT) models were used to examine the temperature profile of the air masses over time (9). This allowed sampling of discrete atmospheric layers and determination of whether the air parcel had interacted with the atmospheric boundary layer or Earth’s surface in the 14 days prior to sampling. Air masses sampled in the troposphere originated from regions over the Gulf of Mexico, while those sampled in the stratosphere had traversed the Atlantic from the vicinity of the West African coast. Each air mass had intersected with severe weather on its trajectory in the United States (Supplemental Material and Methods). Since convective storms can provide sufficient lift to inject air masses from lower altitudes into the free troposphere and stratosphere, regional sources in the continental US for the bacteria cannot be excluded (10).

Initial comparisons based on 16S rRNA gene sequencing of the isolates indicated that various strains were affiliated with the genus *Curtobacterium*, and three had phylogenetically close relationships to *Curtobacterium flaccumfaciens* pv. *flaccumfaciens* (*Cff*). Two *Curtobacterium* isolates, L3-7 and L6-1, were recovered from two separate sampling missions of stratospheric air masses in New Mexico and Texas (2013) (6). An additional isolate, L1-20, was recovered from altitudes ranging from 1.5 to 29 km in New Mexico during 2012. *Cff* is the most economically important plant pathogen of this genus, causing bacterial wilt disease and high crop losses in cultivated legumes; it is currently listed on Europe’s A2 EPPO quarantine list (11). Although most *Curtobacterium* species described to date are associated with plants and leaf litter, *Curtobacterium flaccumfaciens* and *Curtobacterium allii* are recognized plant pathogens (12–14).

In this study, we investigated the plant pathogenicity of three *Curtobacterium* strains that were isolated from aerosol samples collected from the troposphere and stratosphere (Table S1). To evaluate if environmental filtering in the high atmosphere selects suitable tolerance phenotypes, we also examined their resistance to UVR-C and desiccation in comparison to *Cff* reference strains. We analyzed average nucleotide identity (ANI) and digital DNA-DNA hybridization (dDDH) values of whole genome sequence assemblies to determine the phylogenetic relationships among the *Curtobacterium* atmospheric isolates and species for which genome data are available.

**Fig 1:**
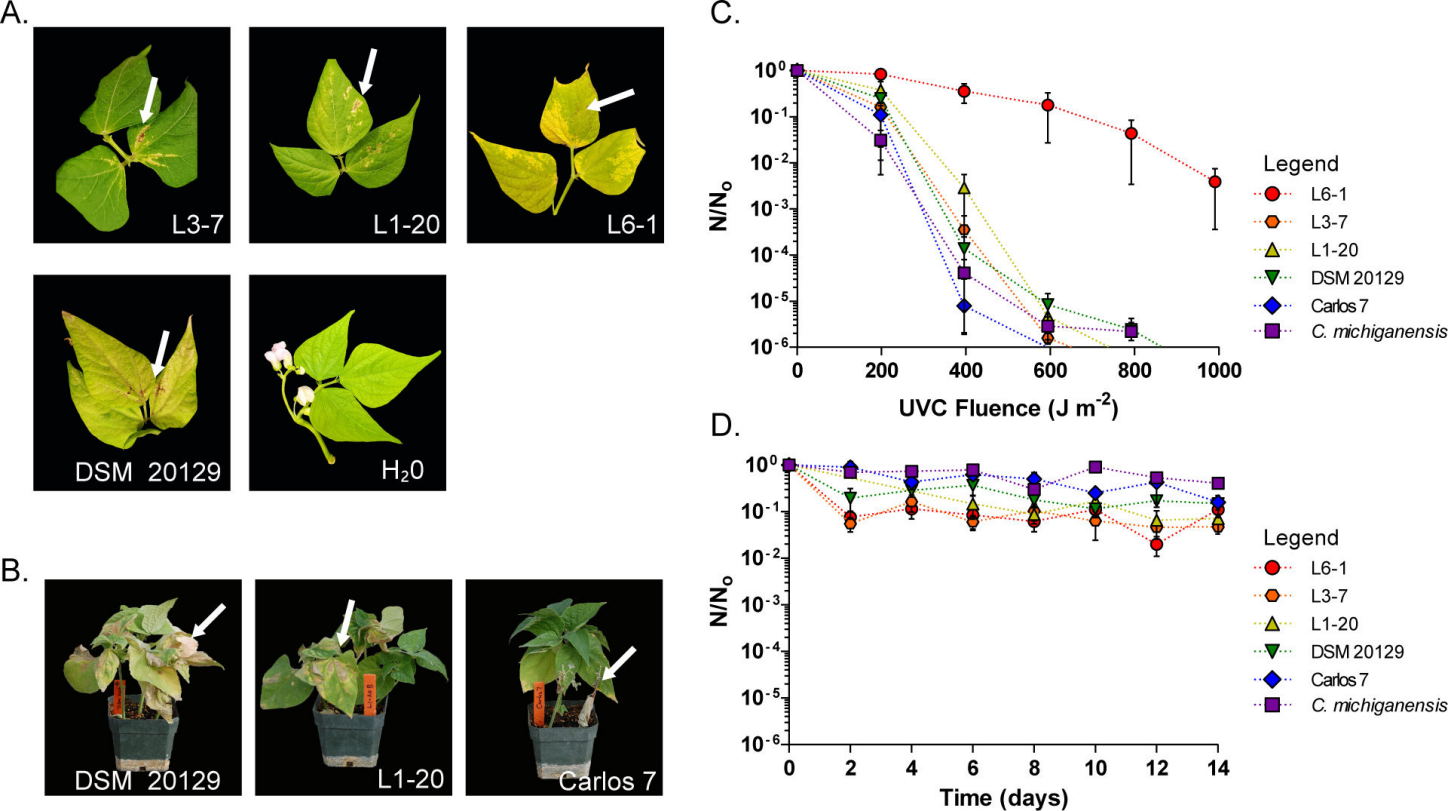
Phenotypic analysis of the stratospheric *Curtobacterium* strains. (**A**) Representative terminal leaves of Borlotto bean plants, 15 days post stem inoculation with stratospheric *Curtobacterium* strains. DSM 20129 and H_2_O serve as positive and negative controls, respectively. (**B**) Representative photos of great northern bean plants inoculated with L1-20, DSM 20129, and Carlos 7 strains. White arrows (**A, B**) indicate symptoms correlating with bacterial wilt. (**C**) and (**D**) Tolerance of *Curtobacterium* strains to UVC radiation and desiccation. Survival curves of each strain after exposure to (**C**) UVC radiation (λ = 254 nm) up to 1000 J m^−2^ and (**D**) desiccation (25–30% RH) for 14 days measured as the surviving number of cells (**N**) at each time point over total number of cells in the unexposed control (**N_0_**). Averages are based on three independent replicates. Error bars represent SEM. *C. michiganensis* CM8704 was used as a control.

Pathogenicity tests were conducted following the method described in the EPPO diagnostic bulletin (15). Three-week-old susceptible bean plants (*Phaseolus vulgaris*, variety Borlotto) were inoculated by stabbing the stem with 2-µL pipette tips that were dipped in a suspension of 2.4 × 10^8^ (colony-forming units) CFU mL^−1^ for each strain. Water was used as a negative control, and the pathotypical *Cff* strain DSM 20129 was used as a positive control. Each strain was tested on a minimum of eight biological replicates per experiment, and the experiment was repeated three times. Bacteria were re-isolated from bean stems above the inoculation zone, and 16S rRNA gene sequencing was performed to confirm that the observed symptoms were caused by the strains inoculated. To corroborate these results, blind studies were performed at a separate site, using a different inoculation technique (16). These experiments included three different dry bean market classes: pinto (cv “Lariat”), a great northern (cv “Orion”), and a kidney bean cultivar. Inoculations consisted of dipping sterile needles into bacterial colonies of 48-hour cultures and inserting them into stems between the first and second node, or just before the first fully expanded trifoliate appeared. The bacterial concentration on needle tips for each plant was estimated to be 5.7 × 10^8^ CFUs (17). Water and a highly virulent field isolate (Carlos 7) from an infected dry bean plant in Nebraska were used as negative and positive controls, respectively, and each test was performed twice (17). The *Curtobacterium* isolates (Table S1) produced symptoms compared to those caused by the pathotypical *Cff* DSM 20129 control strain (Fig. 1A and B). However, none of the strains caused symptoms as severe as the aggressive *Cff* Carlos 7 strain (Fig. 1A).

To explore an association between UVR or desiccation tolerance and pathogenicity of the isolates, we performed survival tests (Supplemental Material and Methods) using the DSM 20129 and Carlos 7 *Cff* strains, as well as the Gram-positive nonspore-forming bacterial plant pathogen *Clavibacter michiganensis* (*Cm*) strain CM 8704A (18) as controls. To examine the tolerance of the populations to UVC radiation, serial dilutions of stationary-phase cultures were spotted onto LB agar plates and exposed to increasing UVC dosage. The number of CFU surviving each UVC radiation dose (N) was used to calculate the surviving fraction. For desiccation tolerance, stationary-phase culture suspensions were incubated in a desiccation chamber, and survival was monitored through CFU counts. All strains tested were moderately tolerant to UVR-C, whereas L6-1 demonstrated a high tolerance (Fig. 1C; Table S2) Near that observed for *Deinococcus radiodurans* R1 (6). Trends in desiccation survival are highly similar among the strains tested (Fig. 1D; Table S2) and indicate that all isolates are highly tolerant to water loss.

**Fig 2:**
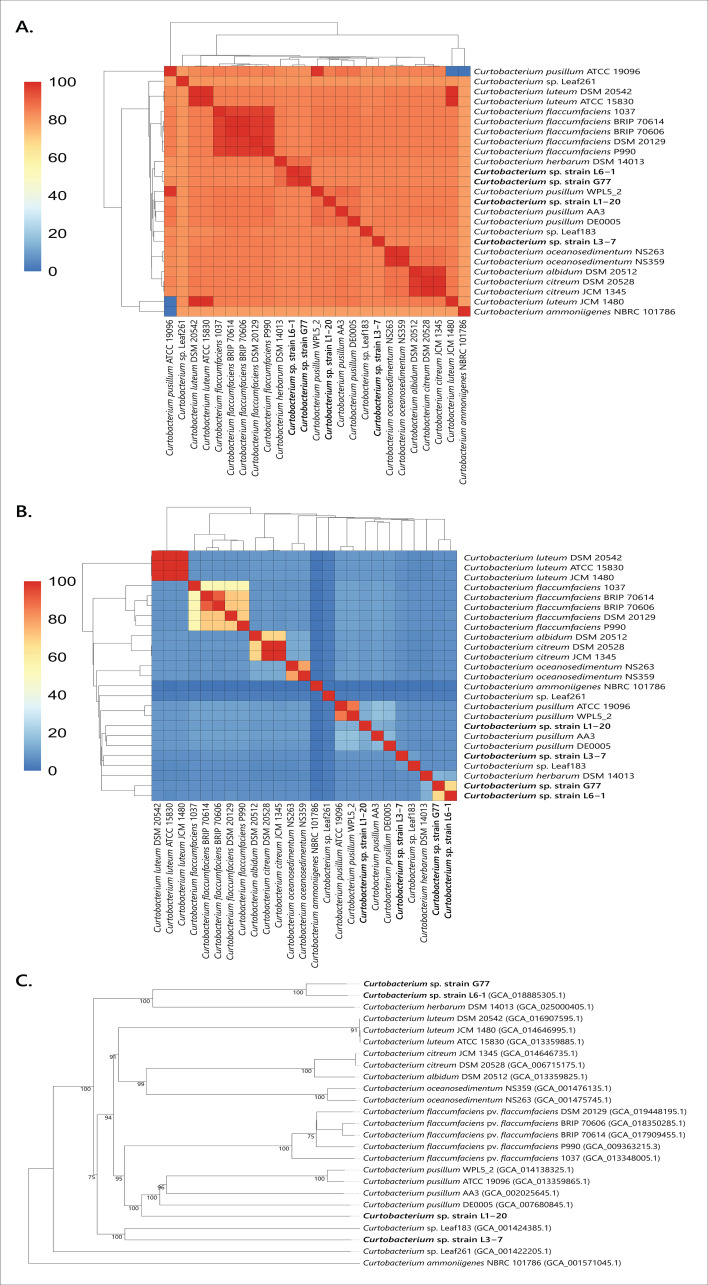
Phylogenetic analysis of atmospheric *Curtobacterium* strains. (**A**) Heatmap and dendrogram of average nucleotide identity (ANI) illustrating the phylogenetic relationships based on the percentage identity shared between the whole genome sequences of atmospheric *Curtobacterium* isolates with *Curtobacterium* strains from GenBank. ANI values greater than 95% represent an accurate threshold for demarcating species for prokaryotes. The dendrograms are produced by single-linkage hierarchical clustering trees from the matrix of pairwise identity results provided in Table S1. (**B**) Heatmap and dendrogram of digital DNA-DNA hybridization (dDDH) pairwise analysis of whole genome sequences of stratospheric *Curtobacterium* isolates and *Curtobacterium* strains from GenBank using the Type Strain Genome Server (TGYS) d4 formula (GGDC formula 2) reflecting the proportion of sequence identity within the homologous parts of the underlying genomes independent of genome length. The dendrograms are produced by single-linkage hierarchical clustering trees from the matrix of pairwise identity results provided in Table S1. dDDH values of greater than 70% are generally accepted as species threshold cutoff (23). (**C**) Whole genome sequence-based tree inferred with FastME 2.1.6.1 from GBDP distances. The branch lengths are scaled in terms of GBDP distance formula *d_5_*. The numbers above branches are GBDP pseudo-bootstrap support values >60% from 100 replications, with an average branch support of 76.6%. The tree was rooted at the midpoint.

The genome sequence and assembly of stratospheric strain L6-1 were generated with PacBio (Pacific Biosciences) sequencing and have been reported elsewhere (GenBank accession CP076544) (19, 20). Draft genome sequences and assembly of isolates L3-7 and L1-20 were conducted with ONT (Oxford Nanopore) long-read technology, with assembly conducted using Flye v2.9.1 and polishing using Medaka v1.8.05 (GenBank accessions CP151472-4). The average nucleotide sequence identity (ANI) was calculated using KBase UI version 2.6.3, with FastANI analysis of imported genomes (21). We curated a small database of available *Curtobacterium* genome assemblies for ANI and dDDH comparison using the Type Strain Genome Server (TYGS) (Table S4A and B) and used the Genome BLAST Distance Phylogeny builder on TYGS to create a whole genome-based tree (22). Based on standard species cutoffs of ANI ≥95% and dDDH ≥70% (23), as well as comparisons with the *Curtobacterium* reference strains, our results are consistent with each isolate representing a distinct *Curtobacterium* species (Fig. 2; Table S4A and B). *Curtobacterium herbarum* DSM 14013 (ANI: 88%) is the closest sequenced relative of isolate L6-1. Strain L1-20 shares a common relative with the *Curtobacterium pusillum* group (ANI: 87%), while the closest relative of strain L3-7 is the sequenced isolate Leaf183 from the unnamed *Curtobacterium* genomospecies 41 (ANI: 86%) (13).

We were curious whether any of the three atmospherically isolated *Curtobacterium* species might have been previously observed in agroecosystems and misidentified as *Cff*. Using a previously published *Cff*-specific diagnostic PCR primer set (24), we screened a collection of sixteen *Curtobacterium* agricultural isolates from various crop species in Iowa, North Dakota, and Nebraska, USA (Table S1). The DSM 20129 control strain and 15 out of 16 *Curtobacterium* isolates were confirmed by PCR as *Cff* (Table S1). As expected, the three atmospheric *Curtobacterium* spp. strains were PCR negative. However, strain G77, isolated from millet in Banner County, Nebraska, also failed to produce a *Cff* diagnostic PCR band. We obtained the genome sequence and draft genome assembly for strain G77 (GenBank accession CP151084) and compared its genome with our sequenced *Curtobacterium* strains. This analysis supported that strain G77 is a member of the same unnamed *Curtobacterium* genomospecies as strain L6-1 (Fig. 2). We propose the name *Curtobacterium aetherium* sp. nov. (Actinomycota, Actinomycetes, Micrococcales, Microbacteriaceae, *Curtobacterium*, *Curtobacterium aetherium,* Etym. of the upper atmosphere) for this species under the SeqCode (25). The L6-1^TS^ genome (GCF_018885305.1) is proposed as the nomenclature type and L6-1 as the reference strain, with *in vitro* and *in vivo* phenotypic characterization supporting its novelty (19). Strain L6-1 has also been deposited in two public culture collections (ATCC TSD-458 and LMG 33684).

The decrease in temperature, air pressure, and relative humidity and the increase in UVR flux constrain habitability with altitude in the atmosphere (6). Environmental filtering for phenotypes facilitating airborne survival may be expected for a phytopathogen with a life cycle in which atmospheric transport serves as an important strategy for disseminating to new locations and hosts. The *Curtobacterium* species we have characterized were recovered from high altitudes in the troposphere and stratosphere and at different times and locations, suggesting that this group is both abundant and physiologically capable of surviving transport in the atmosphere. While a multitude of studies have examined plant pathogen dissemination between fields, or even regionally through lower atmospheric movement (4, 26), intercontinental dispersal has typically been viewed as a low-probability event. Our data demonstrating the presence of *Curtobacterium aetherium* in the atmosphere and agricultural ecosystems provide direct support for the exchange of phytopathogens between terrestrial ecosystems and the high atmosphere.

## Data Availability

All data generated or analyzed during this study are included in this published article and its supplementary information files. Genome assemblies and 16S sequences have been deposited in GenBank, with all of the accessions provided in Table S1.

## SUPPLEMENTAL MATERIAL

Supplemental material spectrum.01774-24-s0001.pdf Supplemental methods.

Supplemental tables spectrum.01774-24-s0002.xlsx Tables S1 to S5.

ASM does not own the copyrights to Supplemental Material that may be linked to, or accessed through, an article. The authors have granted ASM a non-exclusive, world-wide license to publish the Supplemental Material files. Please contact the corresponding author directly for reuse.
