# Supplementary material for: Curtobacterium aetherium sp. nov., a polyextremophilic plant pathogen isolated from the stratosphere
Source: Microbiol Spectr. 2025 Apr 2;13(5):e01774-24. doi: 10.1128/spectrum.01774-24 (PMC12053909; doi:10.1128/spectrum.01774-24)
Supplement: Supplemental material — Supplemental methods. [file spectrum.01774-24-s0001.pdf]

Supplemental Materials and Methods

UVC Radiation Survival Assays

Cultures were grown aerobically in 5 mL LB broth to stationary phase (30°C, 250 rpm) and serially diluted in 10 mM $MgSO_4$. A 10 µL volume of each dilution was spotted in six sections on a square LB agar plate. The plates were then covered with aluminum foil and each section was exposed to increasing dosage of UVC radiation (GE G36T5 UVC Light Bulb; λ = 254 nm; 3.3 W m^{-2}) in 1 min intervals for up to 5 min. The dosage delivered was monitored using a digital radiometer (Solar Light Company, Inc.). The final section of each plate remained unexposed and served as a time zero control (N_0). The cultures were then incubated at 30°C and the number of colony forming units (CFU) surviving each UVC dose (N) was used to calculate the surviving fraction, expressed as the ratio N/N_0. Survival rates are reported as the mean and SEM for three biological replicates. *Clavibacter michiganensis* CM 8704A was used as a control strain. The survival data were fit to exponential decay models of the form $N = N_0e^{-kF}$, where k is the inactivation rate and F is the UVC fluence in J m^{-2}. The values of k, R^2 for the fit models, and LD90 calculated from the models are displayed in Supplemental Table 2. Inactivation rates between the strains were compared by one-way ANOVA and Tukey's post-test. Statistical analyses were performed using the GraphPad Prism 5 software.

Desiccation Survival Assays

Cultures were grown in 1 mL LB broth in a 96-deep well plate to stationary phase (30°C, 900 rpm). Cells were then harvested by centrifugation (5000× g), washed once, and resuspended in 100 µL $MgSO_4$ (10 mM). A 10 µL volume of each cell suspension was spotted into eight 96-well plates and the plates were placed in a desiccation chamber (25-30% RH) for 14 days. Time points were taken every 2 days by removing one plate from the chamber, resuspending and serially diluting the cells in 10 mM $MgSO_4$ and spotting 10 µL of each dilution onto LB agar. Plates were incubated at 30°C until colonies formed. A time zero sample was taken once no visible liquid remained in the wells (~4 hr in desiccation chamber). *Clavibacter michiganensis* CM 8704A was used as a control strain. Survival rates were calculated, and statistical analyses performed as described for the UVC survival assays. Values reported are the mean and SEM for three biological replicates.

DNA extraction, sequencing, and PCR

Curtobacterium strains were cultured from 15% glycerol cryostocks onto LM medium agar plates (10 g/L tryptone, 6 g/L yeast extract, 1.193 g/L KH_2PO_4, 0.6 g/L NaCl, and 0.4 g/L $MgSO_4 \cdot 7H20$, 15g/L agar) for two days at room temperature, then single colonies were picked into LM liquid for overnight culture at 28°C with shaking at 200 rpm. Liquid cultures were normalized to approximately OD_{600} 1.0 with an Eppendorf Biospectrophotometer and 1 ml was pelleted by centrifugation for DNA isolation.

Curtobacterium cell pellets were shipped in Zymo Research DNA/RNA shield to Plasmidsaurus for on-site DNA extraction, whole genome sequencing with Oxford Nanopore Technology followed by custom analysis, assembly, and annotation.

In-house bacterial genomic DNA was extraction was conducted following the Bacteria DNA Isolation CTAB Protocol from DOE Joint Genome Institute (William et al 2012). DNA extracts were checked for concentration and purity on the Eppendorf Biospectrophotometer, then normalized to 20ng/ul for PCR. Amplification of 16s region was conducted with 27F and 1492R primers using Promega GoTaq, with 100ng DNA for amplification; the annealing temp was set to 50°C, and one minute was used for extension time. Bands of approximately 1200 bp were confirmed on 1% agarose TBE gels, and PCR products were cleaned using NEB PCR&DNA Cleanup Kit. Cleaned amplicons were submitted to Eurofins for sequencing with 1492R. Resulting chromatograms were checked via Geneious 10.2.6 before submitting to GenBank for BLAST identification.

To confirm *Cff* identity, the same normalized DNA extracts were PCR amplified with primers CF4 and CF5 to generate a ~200 bp amplicon (Guimaraes et al 2001). The annealing temp was adjusted to 55°C, and extension time was set for 30 sec. Bands were confirmed on 1% agarose TBE gels.

ANI analysis

The genome sequence data were uploaded to the United States Department of Energy Systems Biology Knowledgebase (KBase) (Arkin et al 2018). We used FastANI for alignment-free computation of whole-genome Average Nucleotide Identity (ANI).

TYGS analysis

The genome sequence data were uploaded to the Type (Strain) Genome Server (TYGS), a free bioinformatics platform available under https://tygs.dsmz.de, for a whole genome-based taxonomic analysis (Meier-Kolthoff and Göker 2019). The analysis also made use of recently

introduced methodological updates and features (Meier-Kolthoff et al 2022). Information on nomenclature, synonymy and associated taxonomic literature was provided by TYGS's sister database, the List of Prokaryotic names with Standing in Nomenclature (LPSN, available at https://lpsn.dsmz.de)(Meier-Kolthoff et al 2022).

Determination of closest type strain genomes was done in two complementary ways: First, all user genomes were compared against all type strain genomes available in the TYGS database via the MASH algorithm, a fast approximation of intergenomic relatedness (Ondov et al 2016), and the ten type strains with the smallest MASH distances chosen per user genome. Second, an additional set of ten closely related type strains was determined via the 16S rDNA gene sequences. These were extracted from the user genomes using RNAmmer (Lagesen et al 2007) and each sequence was subsequently BLASTed (Camacho et al 2009) against the 16S rDNA gene sequence of each of the currently 16799 type strains available in the TYGS database. This was used as a proxy to find the best 50 matching type strains (according to the bitscore) for each user genome and to subsequently calculate precise distances using the Genome BLAST Distance Phylogeny approach (GBDP) under the algorithm 'coverage' and distance formula d_5 (Meier-Kolthoff et al 2013). These distances were finally used to determine the 10 closest type strain genomes for each of the user genomes. We added 11 sequences to our analysis to capture the strain diversity. For the phylogenomic inference, all pairwise comparisons among the set of genomes were conducted using GBDP and accurate intergenomic distances inferred under the algorithm 'trimming' and distance formula d_5 (Meier-Kolthoff et al 2013). 100 distance replicates were calculated each. Digital DDH values and confidence intervals were calculated using the recommended settings of the GGDC 3.0 [2,6]. The resulting intergenomic distances were used to infer a balanced minimum evolution tree with branch support via FASTME 2.1.6.1 including SPR postprocessing (Lefort et al 2015). Branch support was inferred from 100 pseudo-bootstrap replicates each. The trees were rooted at the midpoint (Farris 1972) and visualized with PhyD3 (Kreft et al 2017). The type-based species clustering using a 70% dDDH radius around each of the 10 type strains was done as previously described (Meier-Kolthoff and Göker 2019). The resulting groups are shown in Table 1 and 4. Subspecies clustering was done using a 79% dDDH threshold as previously introduced (Meier-Kolthoff et al 2014). The heatmaps for the ANI and dDDH data were created using Rstudio (Team 2015).

Back trajectory analysis

Back trajectories were examined to determine the history of the air masses on the 14 days prior to the collection air sampled from which the various *Curtobacterium* were isolated (Stein et al 2015). Historical storm data from NOAA's National Centers for Environmental Information (https://www.ncei.noaa.gov) was also analyzed for potential impacts on air mass history.

The free troposphere airmass sampled June 2010 primarily originated over the Gulf of Mexico, where waterspouts and marine thunderstorm winds were reported on 9 of the previous 14 days. The three stratospheric airmasses were transported across the Atlantic from the African coast during the period of analysis, with no evidence of vertical descent into the free troposphere. However, severe storm events were recorded near the trajectories for each stratospheric air mass history that was sampled. During May 2013, nineteen tornadoes categorized as EF-3 and EF-4 were observed in Anderson County, TX. Prior to August 1, 2013, De Baca County, New Mexico was classified as experiencing drought conditions. On August 16 and 18, storms produced hail and thunderstorm winds with gusts up to 56 knots within 5 days prior to sampling, respectively. On September 1, 2013, De Baca County returned to being classified as undergoing drought conditions.

Supplemental References

Arkin AP, Cottingham RW, Henry CS, Harris NL, Stevens RL, Maslov S *et al* (2018). KBase: the United States department of energy systems biology knowledgebase. *Nat Biotechnol* **36:** 566-569.

Camacho C, Coulouris G, Avagyan V, Ma N, Papadopoulos J, Bealer K *et al* (2009). BLAST+: architecture and applications. *BMC bioinformatics* **10:** 1-9.

Farris JS (1972). Estimating phylogenetic trees from distance matrices. *The American Naturalist* **106:** 645-668.

Guimaraes PM, Palmano S, Smith JJ, Grossi de Sa MF, Saddler GS (2001). Development of a PCR test for the detection of Curtobacterium flaccumfaciens pv. flaccumfaciens. *Antonie Van Leeuwenhoek* **80:** 1-10.

Kreft L, Botzki A, Coppens F, Vandepoele K, Van Bel M (2017). PhyD3: a phylogenetic tree viewer with extended phyloXML support for functional genomics data visualization. *Bioinformatics* **33:** 2946-2947.

Lagesen K, Hallin P, Rodland EA, Staerfeldt HH, Rognes T, Ussery DW (2007). RNAmmer: consistent and rapid annotation of ribosomal RNA genes. *Nucleic Acids Res* **35:** 3100-3108.

Lefort V, Desper R, Gascuel O (2015). FastME 2.0: A Comprehensive, Accurate, and Fast Distance-Based Phylogeny Inference Program. *Mol Biol Evol* **32:** 2798-2800.

Meier-Kolthoff JP, Auch AF, Klenk HP, Goker M (2013). Genome sequence-based species delimitation with confidence intervals and improved distance functions. *BMC Bioinformatics* **14:** 60.

Meier-Kolthoff JP, Hahnke RL, Petersen J, Scheuner C, Michael V, Fiebig A *et al* (2014). Complete genome sequence of DSM 30083(T), the type strain (U5/41(T)) of Escherichia coli, and a proposal for delineating subspecies in microbial taxonomy. *Stand Genomic Sci* **9:** 2.

Meier-Kolthoff JP, Göker M (2019). TYGS is an automated high-throughput platform for state-of-the-art genome-based taxonomy. *Nature communications* **10:** 2182.

Meier-Kolthoff JP, Carbasse JS, Peinado-Olarte RL, Göker M (2022). TYGS and LPSN: a database tandem for fast and reliable genome-based classification and nomenclature of prokaryotes. *Nucleic acids research* **50:** D801-D807.

Ondov BD, Treangen TJ, Melsted P, Mallonee AB, Bergman NH, Koren S *et al* (2016). Mash: fast genome and metagenome distance estimation using MinHash. *Genome Biol* **17:** 132.

Stein AF, Draxler RR, Rolph GD, Stunder BJ, Cohen MD, Ngan F (2015). NOAA's HYSPLIT atmospheric transport and dispersion modeling system. *Bulletin of the American Meteorological Society* **96:** 2059-2077.

Team R (2015). RStudio: integrated development for R. RStudio. *Inc, Boston, MA* **700:** 879.

William S, Feil H, Copeland A (2012). Bacterial genomic DNA isolation using CTAB. *Sigma* **50**.
